# Dynamics and extreme plasticity of metallic microparticles in supersonic collisions

**DOI:** 10.1038/s41598-017-05104-7

**Published:** 2017-07-11

**Authors:** Wanting Xie, Arash Alizadeh-Dehkharghani, Qiyong Chen, Victor K. Champagne, Xuemei Wang, Aaron T. Nardi, Steven Kooi, Sinan Müftü, Jae-Hwang Lee

**Affiliations:** 1 Department of Mechanical and Industrial Engineering, University of Massachusetts, Amherst, Massachusetts 01002 USA; 2 Department of Physics, University of Massachusetts, Amherst, Massachusetts 01002 USA; 3 Department of Mechanical and Industrial Engineering, Northeastern University, Boston, Massachusetts 02115 USA; 4 United States Army Research Laboratory, Aberdeen Proving Ground, Maryland, 21005 USA; 5 United Technologies Research Center, East Hartford, Connecticut 06108 USA; 6 Institute for Soldier Nanotechnologies, MIT, Cambridge, Massachusetts 02139 USA

## Abstract

Metallic microparticles can acquire remarkable nanoscale morphologies after experiencing high velocity collisions, but materials science regarding the extreme events has been limited due to a lack of controlled experiments. In this work, collision dynamics and nonlinear material characteristics of aluminum microparticles are investigated through precise single particle collisions with two distinctive substrates, sapphire and aluminum, across a broad range of collision velocities, from 50 to 1,100 m/s. An empirical constitutive model is calibrated based on the experimental results, and is used to investigate the mechanics of particle deformation history. Real-time and post-impact characterizations, as well as model based simulations, show that significant material flow occurs during the impact, especially with the sapphire substrate. A material instability stemming from plasticity-induced heating is identified. The presented methodology, based on the use of controlled single particle impact data and constitutive models, provides an innovative approach for the prediction of extreme material behavior.

## Introduction

High-velocity impacts of microparticles often occur in either natural or artificial environments. While sand, ice particles, and minute space debris produce destructive effects on wind turbines^1^, aircraft^2^, and spacecraft^3^, in certain manufacturing methods, such as shot peening^4^, sand blasting^5^, and fluid-jet polishing^6^, speeding microparticles create beneficial properties on the impacted surface of substrate by virtue of impact-induced extreme conditions. Although the impacting microparticles are generally discarded after the engineering processes, in an emerging additive manufacturing method, cold spray (CS), supersonically accelerated metal microparticles are consolidated through extreme plastic deformation^7, 8^. Therefore, particles’ material characteristics attained from the extreme collision event essentially govern the performance of the final macroscopic object. Moreover, as this solid-state consolidation process is typically completed in a very short temporal scale (~10^−8^ s), even nanoscale morphologies of metals can be preserved without significant recrystallization. This aspect is particularly advantageous when nanoscale grains with a large gradient are needed. Indeed, Thevamaran *et al*. recently discovered an extreme gradient nanograined morphology within silver microparticles deformed by supersonic collisions^9^, which is promising for the realization of hard metals with sufficient ductility^10–12^. Therefore, materials engineering using the localized extreme mechanical events can allow us an unconventional metallurgical method to precisely control nanoscale morphologies of bulk metals.

Despite the growing interest of CS as an additive manufacturing method for metals, the materials science underlying the extreme microscopic events in general has not been explicitly understood, primarily because the material response is in a nonlinear, non-equilibrium, and high- strain-rate (HSR) regime. There have been a number of experimental CS studies, including single particle impacts and consolidation^13–15^. However, when the particles are accelerated through a CS system, which is developed for bulk processing, individual particle’s impact velocity, mass, temperature, and shape can only be marginally estimated with various statistical errors. Thus, systematic investigation of the effects of these variables has been limited. Meanwhile, computational modeling has been dedicated to exploring this extreme collision event at various spatial scales^16–20^, providing many insightful results including the predictions of adiabatic shear instability^15^ and localized melting^14, 15^. Regardless of the technical maturity of the computational modeling, however, the persistent open question has been the calibration and validation of the numerical modeling using accurate, repeatable, and systematically obtained experimental reference data, which was produced in the same environments. Consequently, precise understanding of the extreme plastic deformation of metals will be challenging without an experimental breakthrough dealing with the extremely fast mechanical event at the microscale with an extraordinary HSR on the order of 10^6^–10^8^ s^−1^.

**Figure 1:**
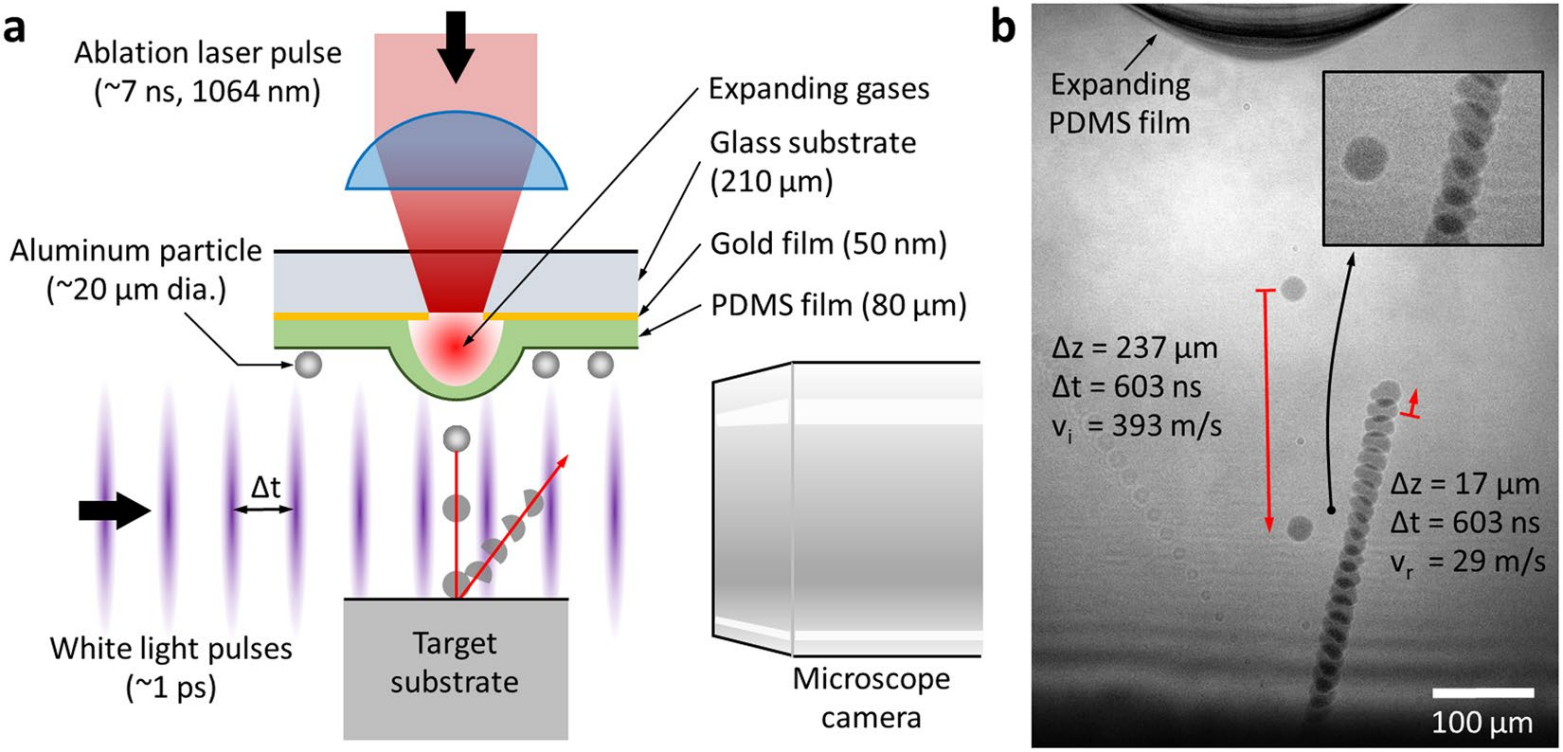
The single aluminum particle impact experiments. (**a**) Scheme of the laser-induced single particle impact experiment. (**b**) 25-times exposure photograph of a colliding and rebounding Al-particle with a 603 ns interval at 1.2 µs after an ablation laser pulse. This inset is a magnified image showing a change of the Al-particle’s shape and a rotational motion of 75,800 revolutions per second.

Our study is primarily focused on the polycrystalline aluminum 6061 alloy (Al), which is composed of 97.5% aluminum and other traceable elements^21^, and is universally used. Its HSR material characteristics differ from pure aluminum^22^. In order to achieve a highly-controlled microscopic single particle collision event, advanced laser induced projectile impact test (α-LIPIT)^23^ parameters are introduced (Fig. 1a) to accelerate Al particles. Due to Al particles’ size distribution (19.3 ± 5.3 µm), the diameter (*D*_0_) of every single Al-particle is measured prior to α-LIPIT, using a long-working-distance optical microscope. The particle is subsequently accelerated by the rapid expanding motion of an 80 µm thick elastomeric film made of cross-linked polydimethylsiloxane (PDMS). The Al-particle can be accelerated to velocities of up to 1,100 m/s without any noticeable laser damage and its impact velocity (*v*_i_) and impact angle (*θ*_i_) with respect to the surface normal direction of a target substrate are quantified using a multiple-exposure photograph (Fig. 1b) taken during the Al-particle’s flight using ultrafast white light pulses. In the acceleration stage of the Al-particle, since the elastomer film is largely compressed by the Al-particles, the film often causes contamination of PDMS on the surface of the Al-particle particularly when *v*_i_ is greater than 800 m/s. Therefore, each particle is checked for PDMS contamination using scanning electron microscopy (SEM). Two distinctive target substrates, sapphire (*G* = 148 GPa)^24^ and Al (*G* = 26 GPa)^24^, are employed for simplified and realistic collision environments, respectively, where *G* is the shear modulus.

**Figure 2:**
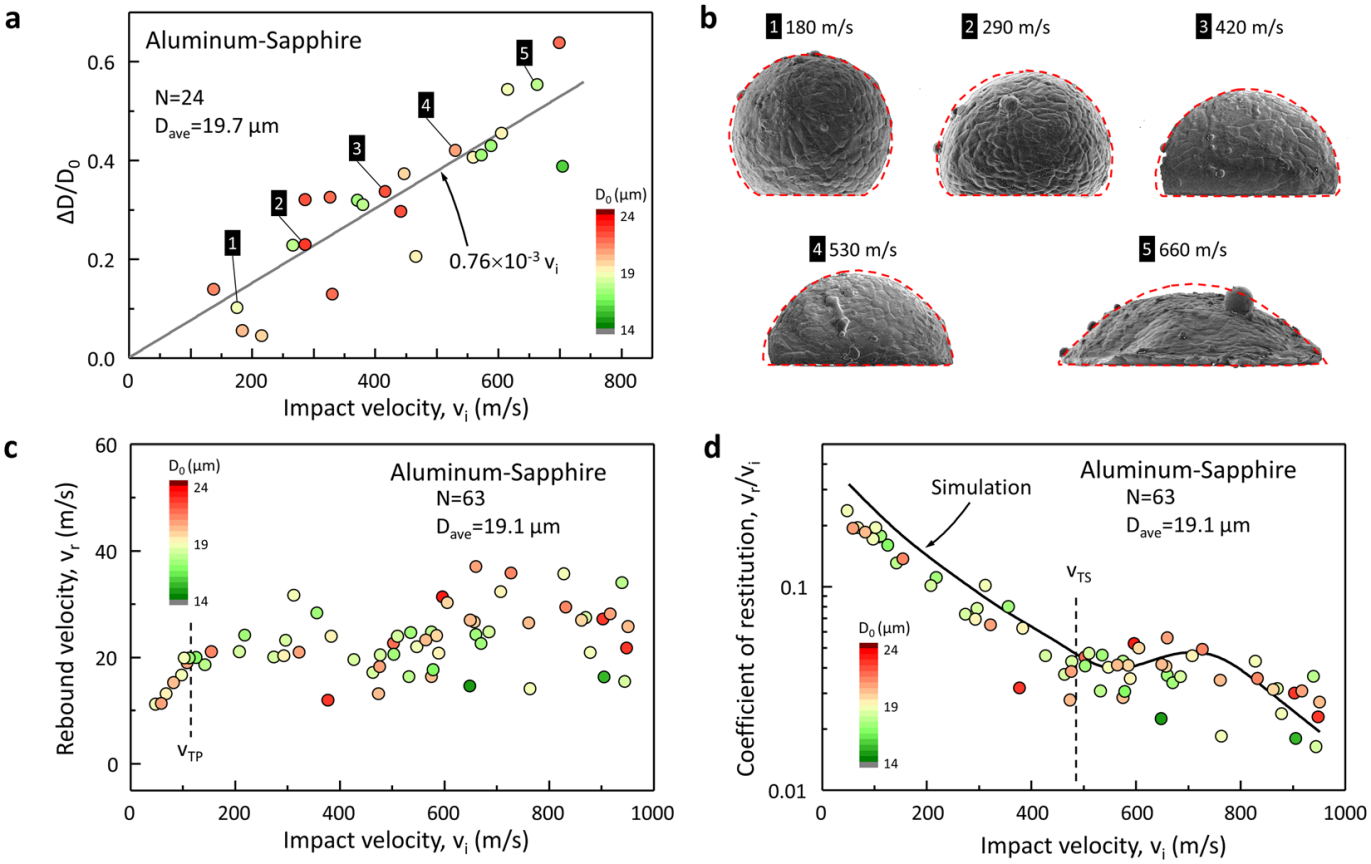
The *in situ* and postmortem characteristics of aluminum particles. (**a**) The specific change of diameter (Δ*D*/*D*_0_) depending on impact velocities is plotted with a linear fitting line. (**b**) Five representative SEM images and outlines of numerically-simulated deformed particles (red dashed lines). (**c**) Rebound velocity of aluminum particles in Al-sapphire collision. (**d)** Coefficient of restitution of aluminum particles in Al-Sap collision with FE simulation results for 19 µm diameter particles.

An Al-particle collision on a sapphire surface (Al-Sap) provides a near-ideal experimental environment for the HSR study of Al-particles without the complex substrate effects, as most plastic deformation occurs in the particle while the substrate remains virtually intact. A total of 63 valid particle collision events are attained over a broad range of *v*_i_ (50–950 m/s). The specific diameter change (Δ*D*/*D*_0_) due to the plastic deformation is linearly proportional to the impact velocities in the range of investigated velocities, where Δ*D* is the change of diameter along the impact direction (Fig. 2a). *D*_0_ does not significantly affect this linear trend. An aggregate strain rate of 6.6 × 10^7^ s^−1^ is estimated, using $\dot{\varepsilon }\cong {v}_{{\rm{i}}}/{\rm{\Delta }}D$, where Δ*D* is given by the relationship, 0.76 × 10^−3^ *v*_i_ *D*_0_, in Fig. 2a. Particle impact is modeled by using three-dimensional large-deformation continuum mechanics with strain-rate dependent and isotropic plasticity. Material heating due to plastic deformation^25^, the effects of temperature on material properties, and the subsequent heat transfer within the material are included in the model. Continuum damage mechanics^26^ is used to account for possible material failure due to accumulation of excessive plastic strain in the material. The interface separation following impact was modeled as a dynamic fracture mechanics problem, by using the cohesive zone modeling^27^. Since the flow (yield) stress of Al, *σ*_Y_, is a strong function of a strain rate, especially when it is greater than 600 s^−1^ ^28^, a constitutive Johnson-Cook (JC) model that accounts for strain rate hardening is used in continuum mechanics based simulations of the impact with the finite element (FE) simulation software ABAQUS^®^. Moreover, a bilinear version of the JC flow stress model that reflects the change in flow stress response is used (Table S1)^29^. The JC model parameters are optimized to match the deformed particle shapes in Al-Sap. The trend of deformed shapes is displayed in Fig. 2b (see Fig. S1 for additional cases). All of the collision events are highly inelastic and 85% or more of initial kinetic energy is dissipated through the collision (Fig. 2c). Rebound velocities linearly increase until *v*_i_ reaches 120 m/s and then fluctuate without a distinctive trend. This is the velocity (*v*_TP_) where the entire material in the particle experiences plastic deformation (Fig. S4). The coefficient of restitution, *C*_r_, a ratio between *v*_r_ and *v*_i_, reveals another transition point (*v*_TS_) near 500 m/s (Fig. 2d) as *v*_r_ starts to increase at *v*_TS_. This transition, also reproduced in the FE simulation using the calibrated JC parameters, is discussed below.

**Figure 3:**
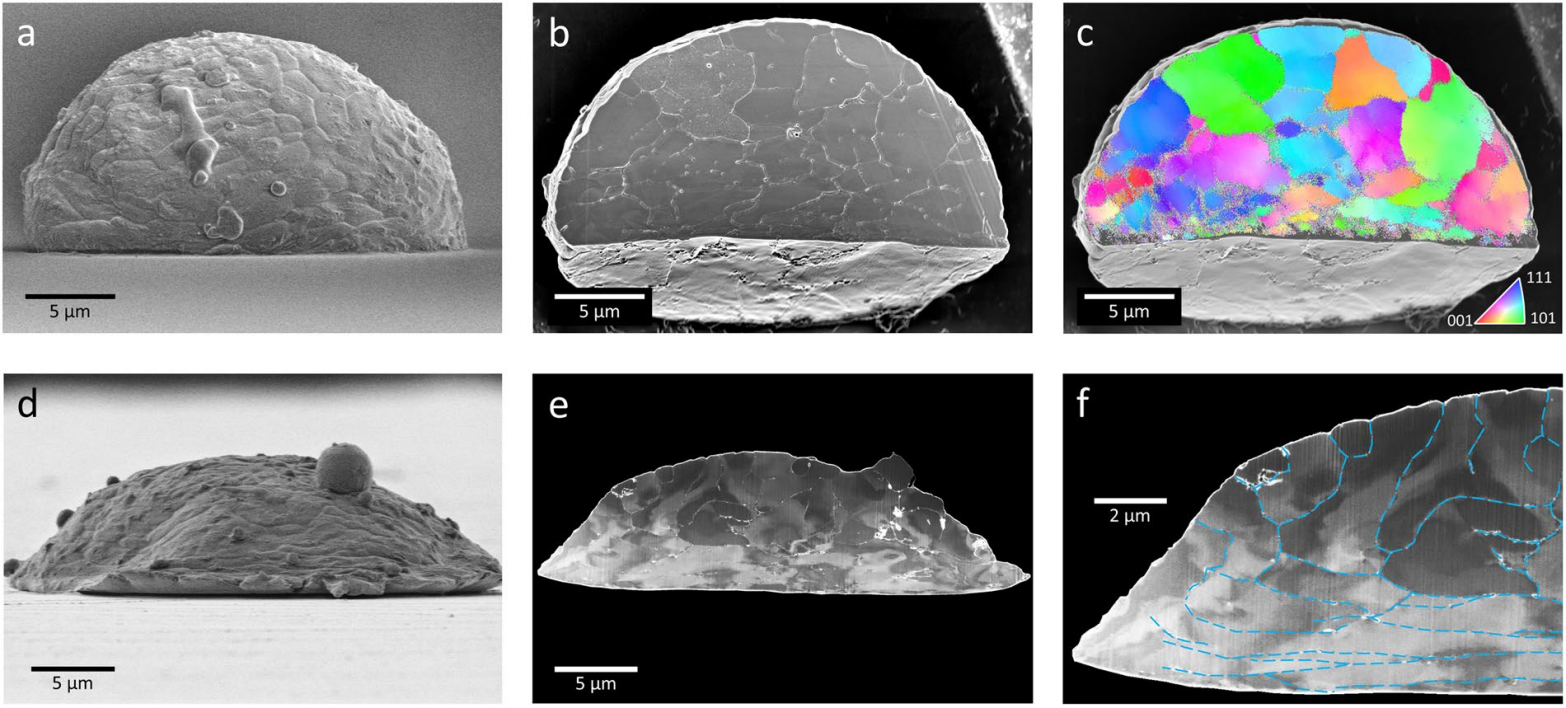
Extreme plastic deformation of the particle after collision to sapphire surface. (**a**) SEM image of the Al-particle deformed by 530 m/s collision to sapphire. (**b**) Contrast-enhanced SEM image after FIB cross section. (**c)** EBSD map of the cross sectional plane in the standard stereographic triangle. (**d**) Side-view SEM image of the Al-particle deformed by 660 m/s collision to sapphire. (**e**) Contrast-enhanced SEM image to show ECC. (**f**) A magnified view of the Al-particle with identified GBs in blue dashed lines.

The Al-particles that impact below 530 m/s maintain their initial spherical shape above the contact region but the Al-particles that impact at 660 m/s or higher evidently display a globally flattened shape in Fig. 2b. To observe the differences in microstructural changes between the two Al-particles deformed 530 m/s (Fig. 3a) and 660 m/s (Fig. 3d), cross sectional studies are carried out using xenon plasma focused ion beam (FIB) milling. The cross-sectioned plane of the 530 m/s particle is additionally etched by brief FIB milling to enhance the contrast of grain boundaries (GBs) (Fig. 3b). The equiaxed grains in the top region indicate that the top region is insignificantly deformed in contrast to the region near the bottom collision face. The electron backscatter diffraction (EBSD) map of the cross sectional plane can visualize residual strain fields within each grain, as well as overall crystallographic information. The crystallographic orientations within a single domain are mainly consistent with limited variation from the primary orientation (Fig. 3c). Note that the unidentified crystallographic orientations at the flattened bottom face, in contrast to the highly confident ones near the top surface, imply that the intrinsic aluminum lattices are too severely distorted to be identified. Compared to the 530 m/s particle, the 660 m/s particle shows a radical change in deformation characteristics, where almost all grains are largely flattened (Fig. 3e). Electron channeling contrast (ECC) arising from crystallographic orientations reveals viscous-fluid-like features crossing over GBs (Fig. 3f). For the collision at *v*_i_ exceeding 550 m/s, we believe that a significant portion of the volume of the Al-particle experienced an extreme pressure higher than *σ*_Y_ and this results in the structural collapse inducing a hydrodynamic state without considerable resistance to shear.

**Figure 4:**
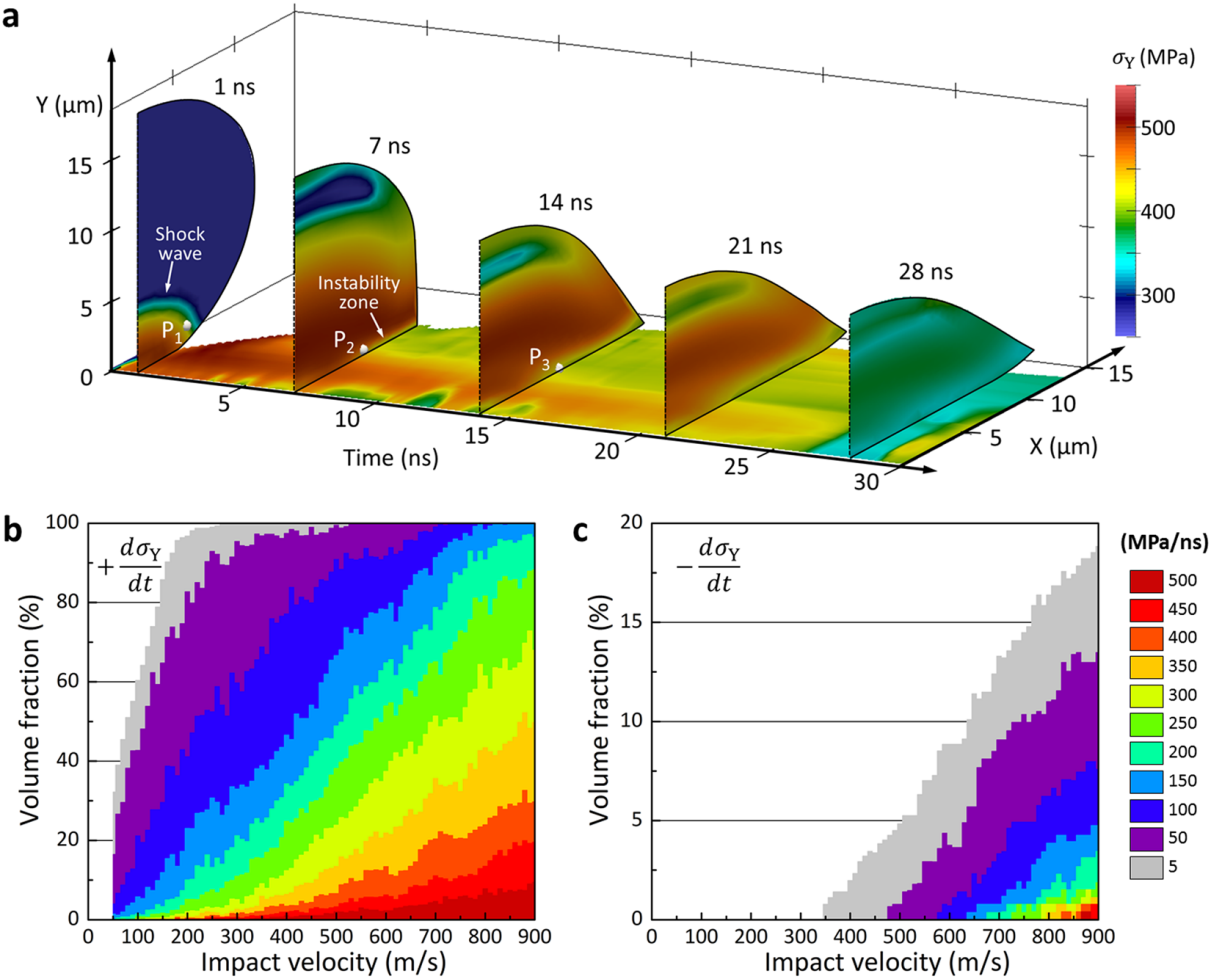
Computed time history of a 19 µm-diameter, spherical Al-particle impacting the sapphire surface. (**a**) Axi-symmetric *σ*_Y_ distribution at a half middle plane of the particle colliding at 550 m/s for first 30 ns of deformation. The history of *σ*_Y_ on the center contact line along the x-axis is shown on the bottom plane. (**b**) The volume fraction of material experiencing positive time-derivatives of *σ*_Y_ as a function of *v*_i_. (**c**) The volume fraction of material experiencing negative time-derivatives of *σ*_Y_ as a function of *v*_i_.

A unique window into the material behavior during deformation is attained by the continuum mechanics simulations of the particle collision. Determination of the model properties for a modified version of the JC model has been the key to provide the description of material behavior in HSR deformation. The simulated deformation and *σ*_Y_ history of the 550 m/s impact of a 19 µm-diameter spherical particle with a sapphire substrate is shown in Fig. 4a, where the points P_2_ and P_3_ are the time-dependent positions of the selected initial position P_1_. During the initial stage of collision, the shock wave propagates at the speed of sound and following HSR deformation rapidly increases the *σ*_Y_. As a result, the local material at P_1_ experiences a substantial increase in *σ*_Y_ from 270 MPa to ~500 MPa in the first ~7 ns of collision (P_2_), followed by an abrupt drop (P_3_) entering the instability zone. Excessive plastic deformation occurs in the instability zone due to a rapid heating in the material, which subsequently causes *σ*_Y_ to drop drastically. As a result, the plastic strain accumulates to very high levels (>3) that cause the material to fail. The *σ*_Y_ histories of other initial material zones are plotted for different *v*_i_ in Fig. S3. For the overall characteristics of the HSR deformation, the time-derivative of *σ*_Y_ at every volume element was monitored and the volume fractions of the particle were quantified as a function of the time derivatives. The volume fraction plot for the positive time-derivatives in Fig. 4b clearly displays that particle’s deformation characteristics are overwhelmingly affected by the material hardening due to large strains and strain rates. For example, more than 90% of the particle’s volume experiences the rapid increase of *σ*_Y_ (*dσ*_Y_/*dt* > 50 MPa/ns) for *v*_i_ > 300 m/s. In addition, a rapid decrease of *σ*_Y_ (*dσ*_Y_/*dt* < −50 MPa/ns) corresponding to the material instability is also found for *v*_i_ > 480 m/s (Fig. 4c), which is correlated with the increase of *v*_r_ for *v*_i_ > *v*_TS_ in the trend of *C*_r_ (Fig. 2d). For *v*_i_ > *v*_TS_, at least part of the particle’s volume (regions-1 and -2 in Fig. S3) experiences fluid like behavior. As a result, we think that, the rate of plastic deformation in the remaining solid is reduced giving the particle more energy to rebound. We also note that the contact area increases due to observed spreading, and also more energy must be stored in the substrate with increasing *v*_i_. The combination of these effects is believed to be responsible for the observed behavior of *C*_r_.

**Figure 5:**
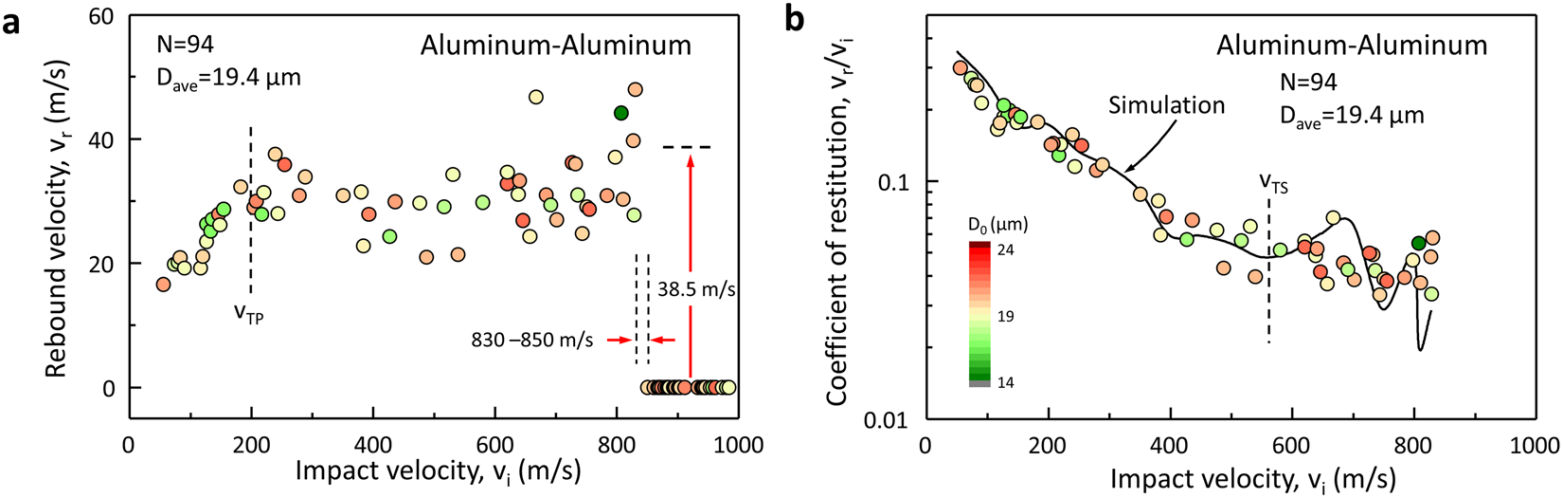
The real-time dynamic characteristics of Al-particles impacting Al-surface. (**a**) Rebound velocity of aluminum particles in Al-Al collision. (**b**) Coefficient of restitution of aluminum particles in Al-Al collision with FE simulation results for 19 µm diameter particles.

Collisions of Al-particle on Al-substrate (Al-Al) are also studied both experimentally and theoretically in the *v*_i_ range of 50–1,000 m/s (Fig. 5a). In contrast to Al-Sap, the Al-substrate dissipates the energy in plastic deformation, but also provides elastic relaxation. Therefore, *v*_TP_ is higher (~200 m/s) than that of Al-Sap due to deformation of the Al-substrate. *v*_r_ (*v*_i_ = *v*_TP_) settles to ~30 m/s due to additional elastic relaxation of Al-substrate. The overall trend of *C*_r_ in Al-Al (Fig. 5b) is similar to that of Al-Sap; however, the transition (*v*_TS_ ~ 600 m/s) is less distinctive compared to the transition in Al-Sap because the deformation of Al substrate reduces the strain rate effects. Above *v*_TP_ and until *v*_i_ approaches *v*_TS_, the slope of *v*_r_ is approximately zero or slightly negative, implying that HSR plastic deformation is capable of absorbing increasing kinetic energy in this range. As we observed in Al-Sap, *v*_r_ starts to grow again, which indicates the onset of material softening and then, abruptly becomes zero. This new transition defines a critical velocity (*v*_c_), 840 ± 10 m/s, determined by the lowest *v*_i_ of bonded Al-particles and the highest *v*_i_ of rebounding Al-particles. Using the average *v*_r_(38.5 m/s) and *D*_0_ (19.8 µm) near *v*_c_, approximately 8.1 nJ of energy was suddenly dissipated and this additional dissipation mechanism is related to the bonded state. The dissipated energy per contact area at *v*_c_ is estimated to be 21 J/m^2^. This includes contributions from both cohesive interfacial energy of aluminum, plastic and elastic deformations (in the substrate) and possible melting of a thin layer of material in the interface. The cohesive interfacial energy of aluminum is on the order of ~1 J/m^2^ ^30, 31^. Therefore, the observed discrete transition of *v*_r_ cannot be attributed to cohesion alone, and first-order transition of Al or melting could be contributing to the observed drop in energy when bonding occurs. Since the dissipated energy of 8.1 nJ is significantly less than the energy (4.4 µJ) required to melt the entire volume of the Al-particle, interfacial melting very localized to the interfacial surface should be considered. Simulations of the Al-Al collisions considering the possibility of cohesion are performed using cohesive zone modeling with ABAQUS^®^ (Fig. 5b). The same optimized JC material model parameters are used for both the particle and the substrate. However, there is a need to take into account the Al-substrate’s HSR behavior separately. Unlike the Al-Sap case, no evidence of material instability is observed. The smallest element volume used in the analysis, (19/25)^3^ µm^3^, could be too large to capture the instability that leads to melting of a very small volume. Nevertheless, by using cohesion energy of 0.5 J/m^2^ and maximum tensile stress of 300 MPa, the critical velocity *v*_c_ of 840 m/s is predicted.

**Figure 6:**
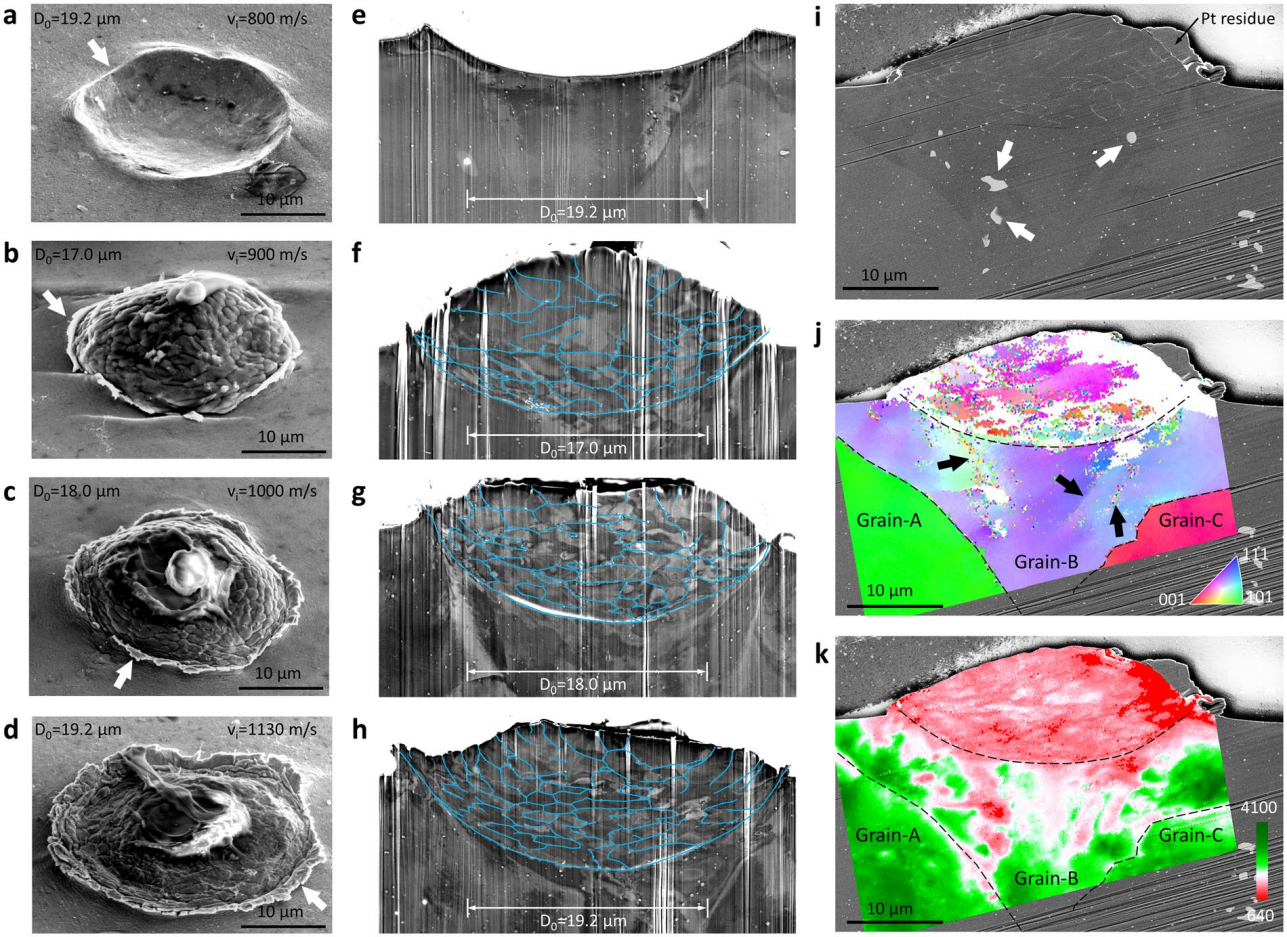
The cross-sectional study of aluminum particles collided on aluminum. (**a–d**) Same-magnification SEM images after 800, 900, 1,000, and 1,130 m/s impacts to Al-substrate. PDMS residues are present on the top surface of each Al-particle. (**e–h**) Contrast-enhanced cross-sectional SEM images of the impact samples in (**a–d**) with identified GBs in blue lines. Each image is scaled to make *D*_0_ the same length. Corresponding original SEM images are shown in Fig. S2. (**i**) A cross sectional SEM image of an Al-particle after a 960 m/s impact to an aluminum surface with *θ*_i_ of 0.6°. (**j**) EBSD orientation map of the deformed particle and substrate in the standard stereographic triangle. (**k**) IQ map of the diffraction patterns.

For the systematic *v*_i_-dependent changes of Al-particles and Al-substrates, Fig. 6 shows the post-impact cross sectional SEM images for four different *v*_i_, 800, 900, 1,000, and 1,130 m/s. In contrast to the circumferential region of the crater (Fig. 6a), one notable difference in the bonding events are the sharply extruded edges (Fig. 6b,c and d). Similar observation is also reported in copper to copper collisions^14^. In conjunction with the abrupt transition of *v*_r_ in Fig. 5a, these extruded features of the edges also support the possibility of interfacial instability. As *v*_i_ becomes higher, in a very confined region of the interface, Al flows much more easily than the rest of the system in a process known as material jetting^16^. This jetting feature is dominantly observed on the substrate side. Because this extruded feature has always been observed when *v*_i_ is larger than *v*_c_, we believe that the material instability in the interface is crucial for the bonding. ECC in the cross sectional SEM images (Fig. 6f–h) reveals extensively distorted grains, both in particles and substrates. In the 900 m/s collision, barely above *v*_c_, the top part of the Al-particle maintained a spherical shape and less variation in the ECC than the bottom region (Fig. 6f). As *v*_i_ is increased to 1,000 m/s, the entirety of the grains of the particle showed rapid variation in ECC due to extensive shear deformation through the entire volume (Fig. 6g). For a 1,130 m/s impact, the particle was further flattened and the plastic flow of materials at the tip of the particle’s edge was completely opposite to the impact direction (Fig. 6h). Although entire GBs were not identified, the deformed grains, which were initially equiaxed, obviously demonstrate the directions of the local plastic flows, as well as the degree of plastic deformation for the different *v*_i_. EBSD mapping of the Al-Al event for 960 m/s was performed to observe the crystallographic information of internal structures of the deformed particles. The cross sectional SEM image shows that entire grains are highly compressed and flattened (Fig. 6i). As seen in the EBSD image of the Al-Sap sample at 530 m/s (Fig. 3c), the crystallographic information of individual grains can be identified. However, crystallographic identification of the bonded particle (Fig. 6j) is very challenging although the local Kikuchi bands^32^ are sufficiently clear. This indicates that the EBSD patterns are not matched with the intrinsic aluminum diffraction patterns, due to extremely distorted atomic lattices arising from complex shear deformations and residual stresses at a scale finer than the spatial resolution of EBSD (~50 nm). The EBSD image also reveals that the bonded particles collided mainly on a single grain of the target substrate, Grain-B, which was aligned close to the (111) plane, and created a large degree of plastic deformation. V-shaped bands (indicated by the arrows in Fig. 6j) of the substrate near the impact center show lattice rotation near GBs under the vertical shock loading^33, 34^. Since the contrast in the image quality (IQ) map (Fig. 6k) constructed from EBSD data is sensitive to local microstructures including phases, strains, and GBs^32^, lower IQ values indicate more extreme deformation of lattices. Indeed, the IQ map showed the highest degree of deformation throughout the entire Al-particle, which also agrees with the observation in Fig. 6g and h. As the bottom right edge of the Al-particle is imbedded below the Al-substrate surface, vertical compression, subsequently followed by lateral compression, results in the highest degree of local deformation within the region. The residual deformation of Al-substrate is relatively lower, and is limited by two GBs and ion-rich intermetallic precipitates (indicated by the arrows in Fig. 6i) in Grain-B, while leaving accumulated strains on the GBs and the precipitates in the IQ map.

In summary, we have demonstrated highly-controlled single Al micro-particle collisions in the extensive range of impact velocities, from 50 to 1,100 m/s. The substantially improved accuracy of numerical simulations using the material parameters, calibrated by the *v*_i_-dependent post-impact dimensions of particles, is confirmed by the excellent agreement between the experimental and simulated dynamic characteristics of the Al particles. The onset of Al particle bonding to Al substrate supports interfacial material instability. In spite of our first successful demonstration, it is also clear that other dynamic effects originating from *D*_0_, *T*, *θ*_i_, grain size, and oxide thickness should be studied to provide further details in the extreme microscopic events. Since the micro-ballistic characterization can be applied to other HSR studies for alloys, polymers, and biomaterials, our approach combined with the constitutive modeling will provide a new avenue in understanding HSR material characteristics of various materials.

## Methods

### Sample preparation

A two-part PDMS kit (Sylgard 184, Dow Chemical) was used with a mixing ratio of 10 to 1 and was spin-coated on an 80 nm thick gold coated microscope cover glass, subsequently followed by curing at 125 °C for 1 hour. The thickness of the cured PDMS film was approximately 80 µm. Aluminum 6061 alloy powders (270 mesh, Valimet, Inc.) were annealed at 230 °C for 1 hour and then sieved to further reduce particle size distribution. The aluminum 6061 particles were mixed with isopropanol and a single drop of the mixture was applied on the PDMS/gold-coated substrate. By placing a small piece of weighing paper on top of the applied mixture, clustering of the aluminum particles due to solvent drying was prevented. Aluminum 6061-T6 target substrate (McMaster-Carr) was used without any additional heat treatment after mechanical polishing using grinding papers and abrasives.

### Microballistic testing

An ablation laser pulse (5–8 ns pulse duration, 1064 nm) was created by using a Nd:YAG laser (Quanta-Ray INDI-40-10-HG, Spectra-Physics). An individual Al-particle was placed near the focal point of the laser ablation on a PDMS/Au/glass substrate (Fig. 1b). A Ti:Sapphire oscillator (Mai Tai HP, Spectra-Physics) continuously provided laser pulses (a pulse duration <100 fs, λ = 750 nm). Since repetition rate of the Ti:Sapphire oscillator provided a reference clock, the pulse repletion rate (79.56 MHz) was precisely measured using a 500 MHz oscilloscope (GDS-3504, Instek). A low-noise and high-quantum-yield digital camera (C11440-22C, Hamamatsu Photonics) is triggered to start image acquisition for 10 ms by a digital delay generator (DG645, Stanford Research System). When the 1064 nm laser pulse was focused at the gold film, the ablation of gold happened and the selected particle was accelerated. During the flight of the Al-particle, the Ti:Sapphire laser pulses were gated by three serially-aligned electro-optical modulators with a high combined extinction ratio greater than 10^7^. For speckle-less and diffraction-suppressed ultrafast photography, the gated laser pulses were converted to white light by a photonic crystal fiber (SCG-800, Newport) exhibiting the supercontinuum conversion^35^. When capturing the rebounding particles, we covered an impact area of specimen with a conic cap having a 100 µm diameter hole at its apex. Al-particles impacted a sapphire substrate though the hole of the conic cover and the impact velocity was measured before entering the conic cover.

### Characterization

For high-speed and gallium-contamination-free sectioning of specimens, xenon plasma FIB milling (Helios PFIB, FEI) was used. To reduce ion damage, platinum (~2 µm thick) was deposited on the surface area to be sectioned prior to FIB. EBSD characterization, cross sectional slices of bonded particles (50 µm × 50 µm × 5 µm) were made and transferred to the other substrate using a nano-manipulator. The transferred slices were further polished using FIB in a different angle to reduce the roughness of sectioned surface. A typical spatial resolution of EBSD mapping was 50 nm.

### Numerical modeling

Mechanics of the particle and the substrate were simulated by using three-dimensional, large deformation, continuum mechanics theory and isotropic plasticity, by using the finite element software ABAQUS^®^ (Dassault Système, USA). Material properties used in the simulations are given in Table S1. Material failure was simulated by using continuum damage failure strain level of 3^26^. Material heating due to plastic deformation, the effects of temperature on material properties and the subsequent heat transfer inside the material were included in the model. The bilinear JC flow stress model^36^ was used to characterize the dependence of the flow (yield) stress *σ*_Y_ on the equivalent plastic strain *ε*_p_, rate of equivalent plastic strain ${\dot{\varepsilon }}_{{\rm{p}}}$ and temperature *T*, as follows^29, 36^,

1 $${\sigma }_{{\rm{Y}}}=(A+B{\varepsilon }_{{\rm{p}}}^{n})[1+C\,{\rm{l}}{\rm{n}}(\frac{{\dot{\varepsilon }}_{{\rm{p}}}}{{\dot{\varepsilon }}_{0}})\,]\,[1-{(\frac{T-{T}_{R}}{{T}_{m}-{T}_{R}})}^{m}]$$

where the parameters *A*, *B*, and *n* are the strain-hardening parameters, *C* controls the strain rate hardening, ${\dot{\varepsilon }}_{0}$ is the reference strain rate, *T*_R_ is a reference temperature, *T*_m_ is the melting temperature of the material, and *m* is the temperature exponent. Recently reported split-Hopkinson bar experiments for Al-6061 show that strain-rate hardening increases dramatically at strain rates higher than 10^3^ s^−1^ ^29^. Manes *et al*. summarized this observation in the context of Johnson-Cook model by using the following bilinear strain rate coefficient^29^,

2 $$\begin{array}{cc}C=\{\begin{array}{cc}{C}_{1}\,{\rm{and}}\,{\dot{\varepsilon }}_{0}=1 & {\rm{if}}\,{\dot{\varepsilon }}_{{\rm{p}}} < {\dot{\varepsilon }}_{{\rm{c}}}\\ {C}_{2}\,{\rm{and}}\,{\dot{\varepsilon }}_{0}={\dot{\varepsilon }}_{{\rm{c}}} & {\rm{if}}\,{\dot{\varepsilon }}_{p} > {\dot{\varepsilon }}_{c}\end{array} & \mathrm{with},\,{C}_{2} > {C}_{1}\end{array}$$

where *C*_1_ and *C*_2_ are coefficients that show the additional increase in the yield stress when ${\dot{\varepsilon }}_{{\rm{p}}}$ is greater than the critical plastic strain rate ${\dot{\varepsilon }}_{{\rm{c}}}$. In this work, these material constants were obtained by least squares curve fitting of the simulation results to the shapes of the deformed particles for the impact velocities as shown in Fig. 2b. The optimization started with the values reported by Manes *et al*.^29^. Following constants were determined *A* = 270 MPa, *B* = 154.3 MPa, *C*_1_ = 2 × 10^−3^, *C*_2_ = 2.9 × 10^−2^, *m* = 1.42 and *n* = 0.239 and ${\dot{\varepsilon }}_{{\rm{c}}}$ = 597 s^−1^ to fit the deformed particle shapes in the 150–700 m/s impact speed ranges very well. Detailed optimized parameters are shown in Table S1.

Mechanics of contact and separation between the particle and substrate are treated independently. During the *compression phase* of the contact, the particle is driven into the substrate only through momentum exchange, as the effects of weak forces (e.g., van der Waals) upon contact are not considered. Under the compressive normal stress and the tangential stress due to Coulomb friction, interfacial bonding occurs by the coupled effects of metallic bonds, localized melting, and/or interfacial mixing. While individual quantification of the various bonding factors is challenging, it is clear that during the compression phase the *interface energy* jumps to a high level relative to the surface energy before bonding. The *separation phase* is identified by development of tensile interfacial stress in the normal direction. The interface behavior during separation is modeled as a cohesive zone^27, 31^. The relationship between the cohesive stress *σ*_*n*_ at the interface and the separation of the two surfaces is represented by a traction-separation relationship. The interface energy is then given by the area under the traction separation curve. We used the triangular traction separation relationship implemented in ABAQUS^®^ with *σ*_*n*_ = 300 MPa and interface energy 0.5 J/m^2^. These two values were obtained by an exhaustive parameter fitting to the experimental results presented in Fig. 5b.

## Electronic supplementary material

Supplementary Information
