# Supplementary material for: Dynamics and extreme plasticity of metallic microparticles in supersonic collisions
Source: Sci Rep. 2017 Jul 11;7:5073. doi: 10.1038/s41598-017-05104-7 (PMC5505959; doi:10.1038/s41598-017-05104-7)
Supplement: Supplementary file 1 — Supplementary Information [file 41598_2017_5104_MOESM1_ESM.pdf]

SUPPLEMENTARY INFORMATION

# Dynamics and extreme plasticity of metallic microparticles in supersonic collisions

Wanting Xie[1,2], Arash Alizadeh-Dehkharghani[3], Qiyong Chen[3], Victor K. Champagne[4], Xuemei Wang[5], Aaron T. Nardi[5], Steven Kooi[6], Sinan Müftü [3*], and Jae-Hwang Lee[1*]

[1]Department of Mechanical and Industrial Engineering, University of Massachusetts, Amherst, Massachusetts 01002, USA.
[2]Department of Physics, University of Massachusetts, Amherst, Massachusetts 01002, USA.
[3]Department of Mechanical and Industrial Engineering, Northeastern University, Boston, Massachusetts 02115, USA.
[4]United States Army Research Laboratory, Aberdeen Proving Ground, Maryland 21005, USA.
[5]United Technologies Research Center, East Hartford, Connecticut 06108, USA.
[6]Institute for Solider Nanotechnologies, MIT, Cambridge, Massachusetts 02139, USA.
*E-mail: leejh@umass.edu; s.muftu@neu.edu

## Supplementary Figures

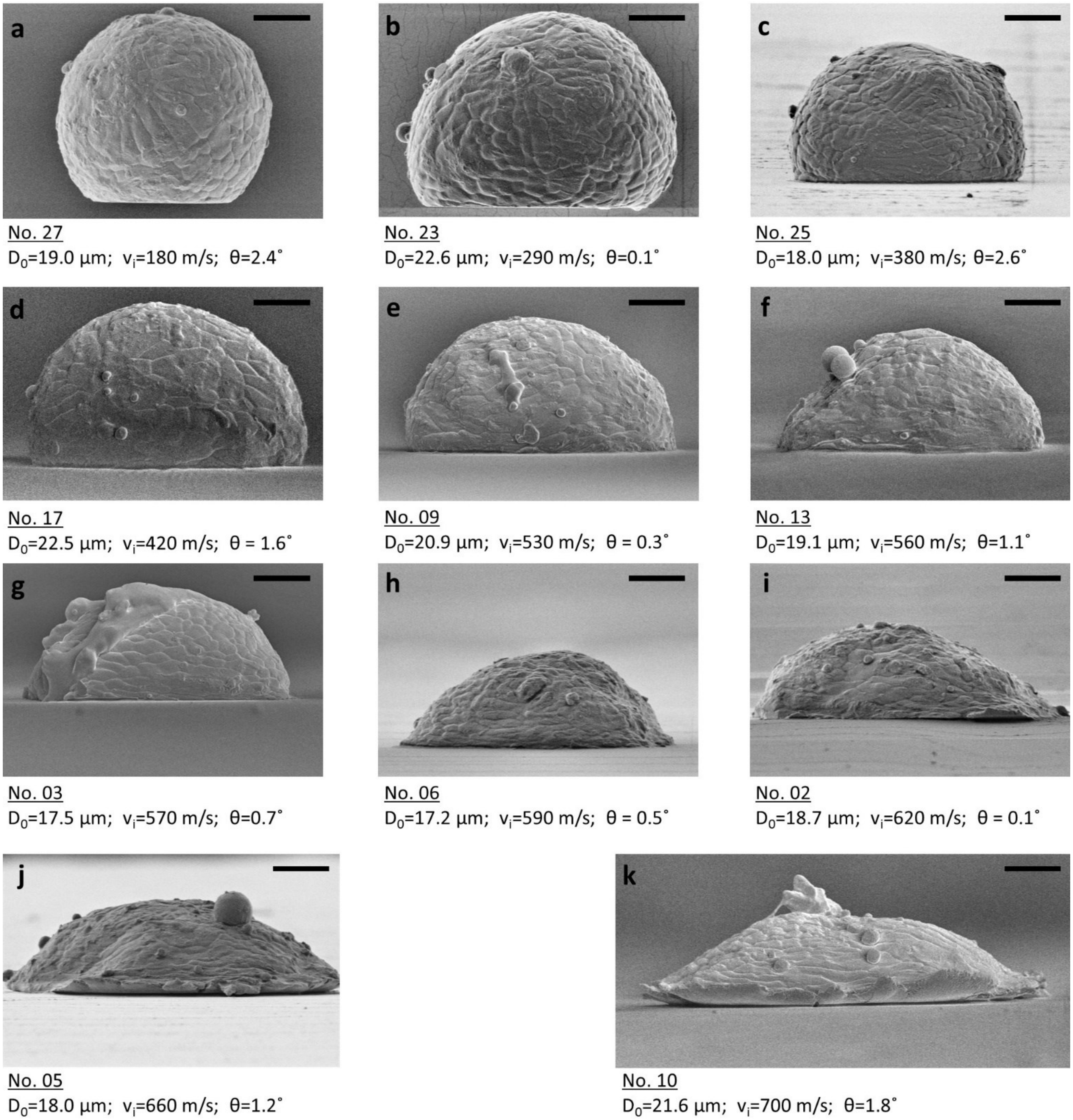


**Figure S1 | Deformed aluminum 6061 alloy particles after collisions to sapphire surface.** (a) – (k) Side view SEM images of deformed particles are shown with their initial diameters, impact velocities, and impact angles. All scale bars are 5 µm.

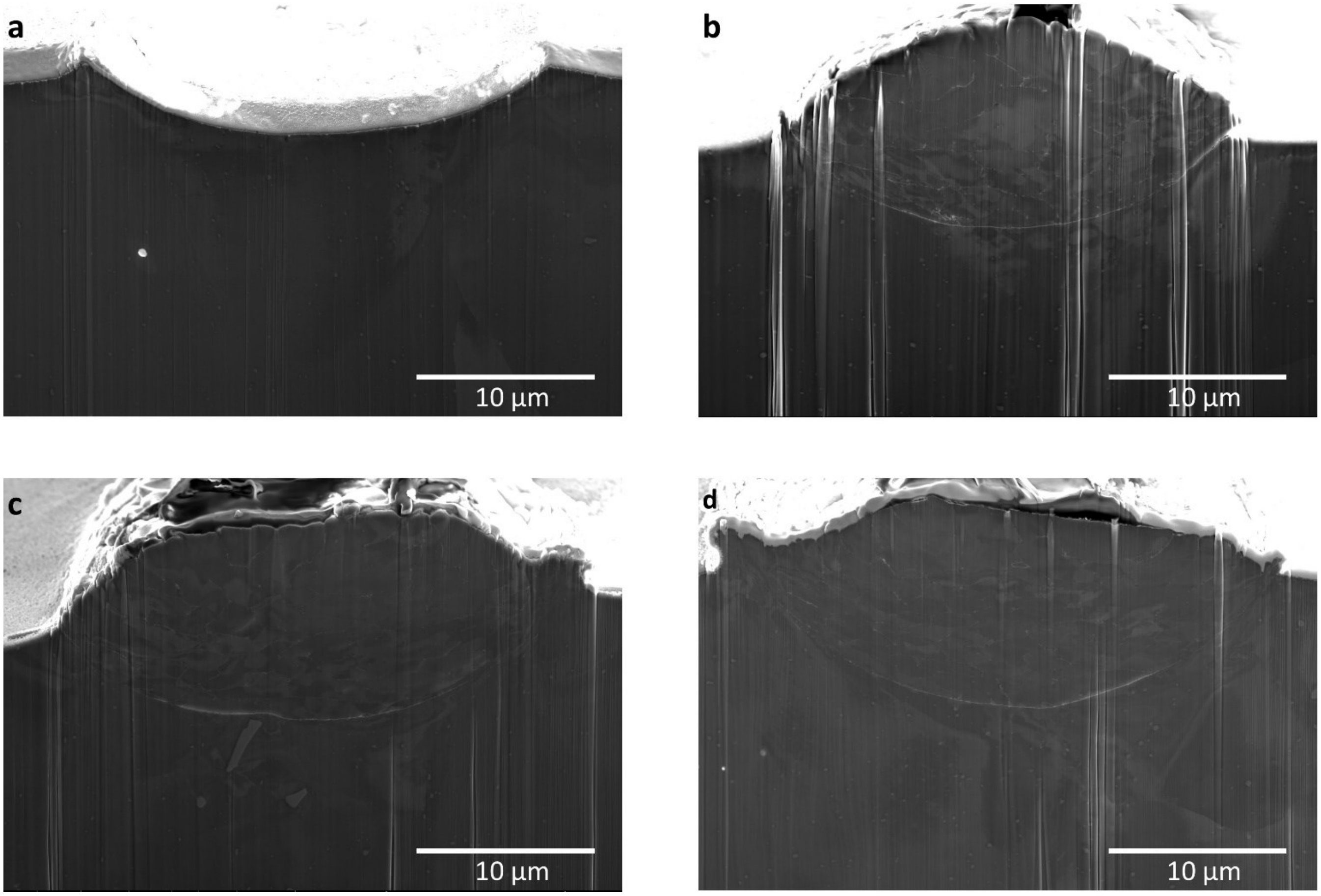


**Figure S2 | Deformed aluminum 6061 alloy particles after collisions to aluminum 6061 surface.** (a) – (d) The original cross-sectional SEM images of **Fig. 5e-h**.

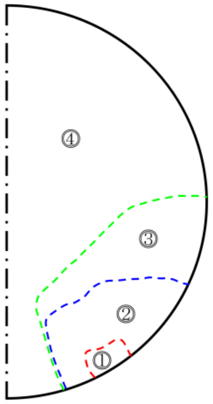


a)

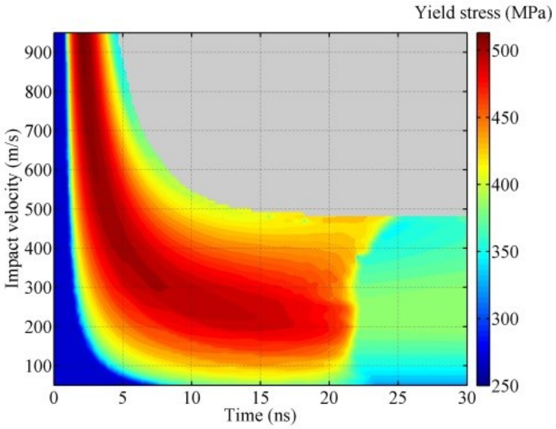


b) Region-1

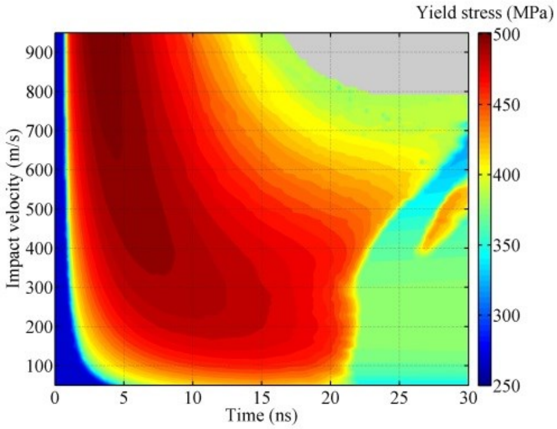


c) Region-2

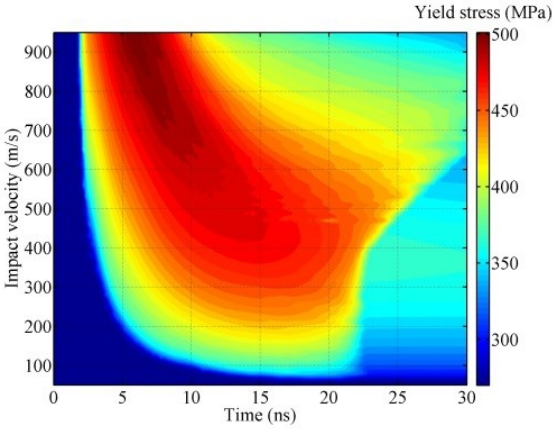


d) Region-3

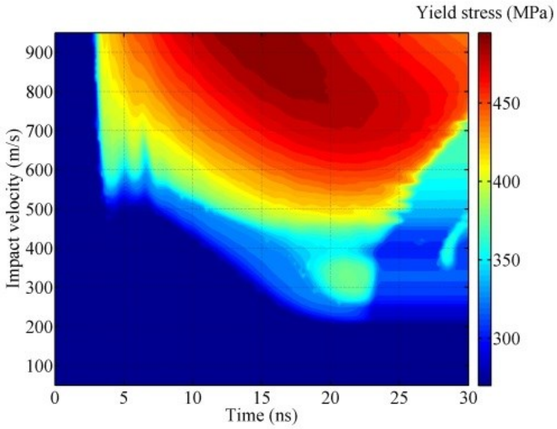


e) Region-4

**Figure S3 | Yield stress variation with time and impact velocity in the four characteristic regions of the particle.**

**Fig. S3a** shows four regions in the particle that exhibit different material responses from this point of view. The material in regions-1 and -2 experiences significant softening during impact, where the instability occurs after 500 m/s in region-1 and 800 m/s in region-2 (**Fig. S3b, c**). In regions-3 and -4, the deformation is predicted to be less severe and no instability is observed, albeit in region-3 some softening is predicted above 700 m/s (**Fig. S3d, e**).

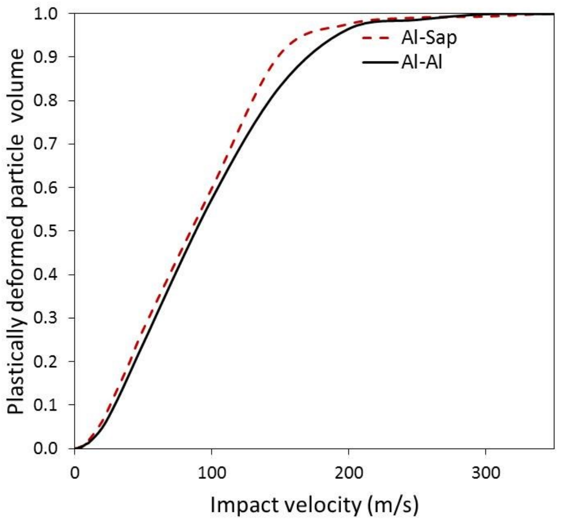


**Figure S4 | Plastically deformed volume of the particle normalized with respect to the particle volume, shown as a function of impact velocity for both Al-Al and Al-Sap impacts.**

**Table S1**: Material properties for the materials used in finite element simulations.

| Properties | Parameter | Value | | Unit |
|---|---|---|---|---|
| | | Al-6061 | Sapphire | |
| General | Density, $\rho$ | 2,700[a] | 3,980 | kg/m$^3$ |
| | Specific heat, $C_P$ | 1,009[a] | 755[a] | J/kg K |
| | Thermal conductivity | 154[a] | 33[a] | W/m K |
| | Thermal expansion | 22.3[a] | 4.6[a] | $10^{-6}$/K |
| | Melting temperature, $T_m$ | 925 | – | K |
| | Inelastic heat fraction, $\beta$ | 0.9 | – | |
| Elastic | Elastic modulus | 69.11[a] | 416[a] | GPa |
| | Poisson's ratio | 0.331[a] | 0.231 | |
| Plastic (Johnson-Cook plasticity model) | $A$ | 270 | – | MPa |
| | $B$ | 154.3 | – | MPa |
| | $n$ | 0.239 | – | |
| | $C_1$, $C_2$, | $2\times10^{-3}$, $29\times10^{-3}$ | – | |
| | [illegible] | 597.2 | – | |
| | $m$ | 1.42 | – | |
| | Reference strain rate | 1 | – | 1/s |
| | Reference temperature | 293 | – | K |
| Material failure | Failure strain | 3 | – | |

[a] Temperature dependent properties. The values at room temperature (293 K) are shown. Data is taken from MPDB software.[1]

1 *Material Property Database ver. 7.99*. (JAHM Software, Inc., 2016).
